# Recent Advances in Structural Studies of Cytochrome *bd* and Its Potential Application as a Drug Target

**DOI:** 10.3390/ijms23063166

**Published:** 2022-03-15

**Authors:** Thorsten Friedrich, Daniel Wohlwend, Vitaliy B. Borisov

**Affiliations:** 1 Institut für Biochemie, Albert-Ludwigs-Universität Freiburg, D-79104 Freiburg, Germany; friedrich@bio.chemie.uni-freiburg.de (T.F.); wohlwend@bio.chemie.uni-freiburg.de (D.W.); 2 Belozersky Institute of Physico-Chemical Biology, Lomonosov Moscow State University, Leninskie Gory, 119991 Moscow, Russia

**Keywords:** molecular bioenergetics, inhibition, electron transport chain, membrane protein, terminal oxidase, cytochrome oxidase, enzyme structure

## Abstract

Cytochrome *bd* is a triheme copper-free terminal oxidase in membrane respiratory chains of prokaryotes. This unique molecular machine couples electron transfer from quinol to O_2_ with the generation of a proton motive force without proton pumping. Apart from energy conservation, the *bd* enzyme plays an additional key role in the microbial cell, being involved in the response to different environmental stressors. Cytochrome *bd* promotes virulence in a number of pathogenic species that makes it a suitable molecular drug target candidate. This review focuses on recent advances in understanding the structure of cytochrome *bd* and the development of its selective inhibitors.

## 1. Introduction

The terminal oxidase cytochrome *bd* (EC 7.1.1.7) is so far found only in the electron transport chains (also known as the respiratory chains) of bacteria and archaea [1]. Notably, even some microorganisms considered to be strict anaerobes possess the active *bd* oxidase to respire, which enhances their growth [2,3]. Cytochrome *bd* reduces molecular oxygen to water at the expense of the concomitant oxidation of species-specific quinols such as ubiquinol, menaquinol, and possibly plastoquinol [4,5]. The chemical energy released in this redox reaction is conserved in the form of a proton motive force across a proton-impermeable prokaryotic membrane [6,7,8]. The proton electrochemical gradient is used by the cell to drive ATP synthesis and to do other useful work. The *bd* oxidase is made up of two to four polypeptide chains, depending on the species. The catalytically active subunit bears the three heme prosthetic groups, *b*_558_, *b*_595_, *d*, and the Q-loop. [9,10,11,12,13,14,15]. The latter is a quinol-binding domain located in the hydrophilic loop region between the transmembrane helices 6 and 7. Until recently, the classification of the cytochrome *bd* family was based on the size of the Q-loop. Accordingly, it was divided into two subfamilies: L (long Q-loop) and S (short Q-loop) [16,17]. However, very recently the phylogeny of the *bd* family has been reassessed by Murali et al. [1]. As a result, three families and several subfamilies within the cytochrome *bd* superfamily have been identified (see [1] for details). Heme *d* is the site for O_2_ binding, usually with a very high affinity, and activation for reduction to 2H_2_O [18,19]. Heme *b*_595_ facilitates heme *d* to carry out the catalytic reaction, and there is significant interaction between these hemes [20,21,22,23,24]. Cytochrome *bd* is phylogenetically unrelated to the heme-copper oxidases and alternative oxidases, the two other groups of terminal oxidases which use O_2_ as the final electron acceptor [16,25,26,27,28,29,30,31,32,33,34,35,36,37,38,39,40,41,42,43,44,45,46,47]. Unlike the heme-copper oxidases, the *bd* enzyme does not pump protons and generates the proton motive force solely by transmembrane charge separation [48,49,50,51]. The role of most heme-copper enzymes is likely limited to energy production, while cytochrome *bd* has other, alternative functions [52]. The ability of cytochrome *bd* to consume O_2_ at high rates not only enables oxidative phosphorylation but also gives protection to O_2_-labile proteins in a wide range of oxygen tensions, including full aerobiosis [53]. The *bd* enzyme plays the O_2_-scavenging role in anaerobes/aerotolerant bacteria, such as *Desulfovibrio vulgaris* Hildenborough, as shown by Ramel and coworkers [54,55]. The *bd* oxidase is necessary for extracellular matrix production and biofilm development [56]. Induction of cytochrome *bd* is part of the bacterial defense mechanisms against the effects of heat stress [57]. The *bd* oxidase helps microorganisms to survive in the presence of various environmental stressors including nitric oxide (NO) [56,58,59,60,61,62,63,64], ammonia [65], nitrite [66,67], sulfide [68,69,70,71,72], chromate [73], and cyanide [68]. Cytochrome *bd* can also directly decompose hydrogen peroxide [74,75,76] and peroxynitrite [77]. Thanks to this specific catalytic capacity, the *bd* enzyme contributes to bacterial resistance against these harmful oxygen and nitrogen reactive species produced by the host immune system to eliminate microbial invaders. Furthermore, cytochrome *bd* is involved in bacterial protection against antibiotic-induced stress [78,79,80]. Probably due to these unique properties, the *bd* oxidase supports virulence in different bacterial pathogens (see [52] and references therein). Since the *bd* enzyme is not encoded by the human and animal genomes, it can serve as an attractive and promising therapeutic target for next-generation antimicrobials. The first three-dimensional structure of the heme-copper counterpart, cytochrome *c* oxidase, was published in 1995 [81,82]. It took more than 20 years to resolve the first three-dimensional structure of the *bd* oxidase from *Geobacillus thermodenitrificans* [9]. The single-particle cryoelectron microscopy (cryo-EM) structures of cytochromes *bd* from *Escherichia coli*, *Mycobacterium smegmatis*, and *Mycobacterium tuberculosis* have been reported over the past three years, with four structural papers published in 2021 [10,11,12,13,14,15]. The emergence of cytochrome *bd* structures has spurred the search for effective and selective inhibitors of this type of enzyme which could become new antibacterial agents. This review focuses on the most recent advances in the structural analysis of the terminal oxidase cytochrome *bd* and the search for its inhibitors.

## 2. Recent Advances in the Structural Biology of Cytochrome *bd* Oxidases

**Figure 1:**
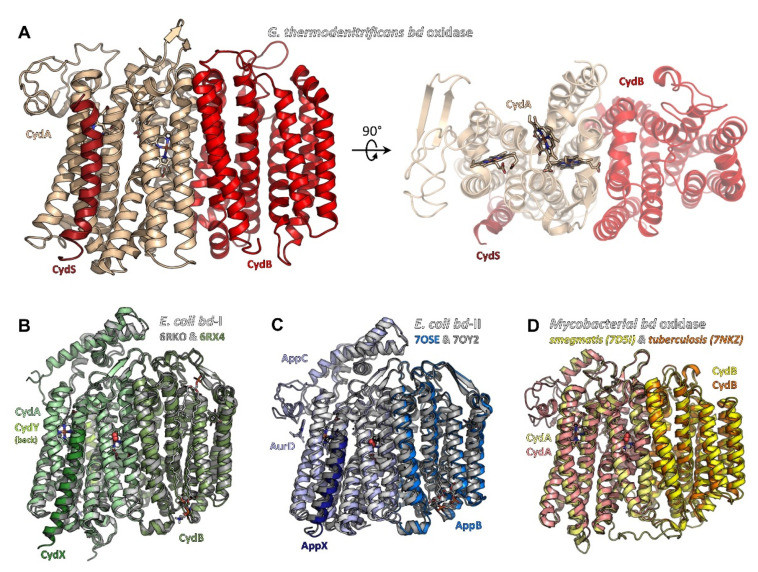
Structures of bacterial cytochrome *bd* oxidases. (**A**) *bd* oxidase of *G. thermodenitrificans* (pdb ID 5DOQ) is composed of subunits CydA (light beige), CydB (red), and CydS (dark red). The heme groups are located in subunit CydA. (**B**) *E. coli bd*-I (pdb ID 6RKO in grey tones, pdb ID 6RXO in green colours) comprises four subunits, termed CydA, CydB, CydX, and CydY. While CydA, CydB, and CydX have homologues in *G. thermodenitrificans*, CydY is exclusive for *E. coli bd*-I. (**C**) *bd*-II oxidase from *E. coli* (pdb ID 7OSE in blue colours, pdb ID 7OY2 in grey tones) is built by subunits AppC (homologue to CydA), AppB (homologue to CydB), and AppX (homologous to CydS/X). 7OSE has been solved with the inhibitor aurachin D (AurD) bound to the Q-loop. (**D**) The mycobacterial *bd* oxidase (*M. smegmatis*, pdb ID 7D5I, in yellow colours, *M. tuberculosis*, pdb ID 7NKZ, in orange and salmon) consists of only two subunits, CydA and CydB.

High-resolution structures of oxidases from the class of heme-copper oxidases (HCO) [83] and alternative oxidases (AOX) [84] have been known for many years. By contrast, structures of the last class of oxidases, namely the *bd* oxidases, have only been obtained over the last few years. On the basis of sequence comparisons, secondary structure predictions and biochemical analysis [16], it was concluded that the class of *bd* oxidases shares no structural similarity with the HCO and AOX classes. Originally, it was assumed that the *bd* oxidases simply consisted of the two large subunits, CydA and CydB. Furthermore, sequence comparison did not reveal that these two subunits share the same fold (see below). The presence of one or two additional small proteins called CydX (or CydS) and Cyd Y (or CydH) in some *bd* oxidases was also not known. When the structures were finally published, it turned out, quite surprisingly, that the heme groups were not arranged in a linear fashion as was originally assumed, but rather in a triangular pattern. In that triangle, the distance between the central iron ions varies between approximately 11 and 19 Å. Accordingly, there is no similarity between the active sites of the *bd* oxidases and that of the HCOs, which have a distance between the heme iron and the copper centre of about 4 to 5 Å, nor to that of the AOXs, with a non-heme di-iron distance of about 3.5 Å. Thus, the active site of *bd* oxidases is a single *d*-type heme and not a so-called ‘binuclear centre’ as in HCOs and AOXs. In the following, we will concentrate on the comparison of *bd* oxidases from different organisms (Figure 1).

Structural analyses of bacterial cytochrome *bd* oxidases by means of X-ray crystallography (*Geobacillus thermodenitrificans*, pdb IDs 5DOQ & 5IR6, [9]) and cryo-electron microscopy (cryo-EM) (*Escherichia coli* *bd*-I, pdb IDs 6RKO [11] and 6RX4 [10]; *Mycobacterium smegmatis*, 7D5I [12]; *Mycobacterium tuberculosis*, 7NKZ [13] and E. coli *bd*-II, 7OSE [14] and 7OY2 [15]) have shed light on the architecture of this class of oxidases.With the exception of the *Geobacillus* structures 5DOQ and 5IR6 that were obtained by X-ray crystallography, all structural data are based on cryo-EM. Resolutions range from 3.8 Å (5IR6, X-ray crystallography) to 2.5 Å (7NKZ, cryo-EM), providing near-atomic details (for a detailed discussion of optical resolutions in X-ray crystallography and cryo-EM, we suggest the work of Dubach and Guskov [85]). This work has unveiled common features shared by all members of the *bd* oxidase class that are distinctly different from the members of the HCO and AOX classes. At the same time, however, the *bd* oxidase structures also disclosed intriguing peculiarities that seem to reflect the differing demands on these enzymes in dependence of the metabolic context, in which they are active. It appears that the *bd* oxidases found in different bacteria represent a variation of a common theme: beyond the shared, basic architecture they differ in the number of the additional subunits, in the arrangement of the hemes and in the position of the quinol binding site.

### 2.1. Common Architecture of Cytochrome bd Oxidases

**Figure 2:**
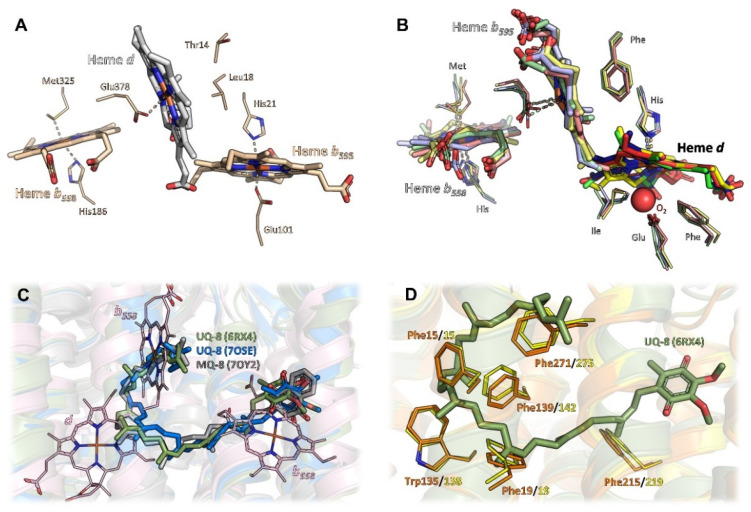
Triangular heme arrangement in CydA/AppC and equivalent positions in CydB/AppB. (**A**) In *G. thermodenitrificans*, hemes *b*_558_ and *b*_595_ (light beige sticks) are found in a plane, while heme *d* sits orthogonally on top (grey sticks). Here, the orthogonal heme is the active site. Axial heme ligands of CydA are shown as thick lines. Thr14 and Leu18 form a hydrophobic roof above heme *d*. (**B**) The heme arrangement in *E. coli bd*-I (green sticks), *E. coli bd*-II (blue sticks), as well as the mycobacterial *bd*s (*M. smegmatis* in yellow sticks, *M. tuberculosis* in red sticks) is conserved and differs from *G. thermodenitrificans* in the relative position of hemes *b*_595_ and *d*. Heme *b*_595_ is now orthogonally placed with respect to the plane and replaces heme *d* (bold colours), which in turn resides in the plane with *b*_558_ (light colours). Nonetheless, heme *d* (bold colours) remains the active site with molecular oxygen (red spheres) being found as axial ligand in pdb ID 7NKZ (*M. tuberculosis*). (**C**) Superposition of *E. coli bd*-I subunit CydA with subunits CydB/AppB of *E. coli bd*-I and *bd*-II. The Q-loop was removed from CydA prior to superposition. In CydB/AppB, either ubiquinone-8 or menaquinone-8 occupies the position corresponding to the heme groups in CydA. (**D**) Mycobacterial *bd*s do not employ a quinone in subunit CydB to fill the equivalent positions of the heme groups, but instead utilise aromatic side chains (shown as thick lines, *M. smegmatis* in orange, *M. tuberculosis* in yellow) to seamlessly fill the available space. Ubiquinone-8, as found in *E. coli bd*-I (6RX4), is given as reference in olive-green sticks.

Overall, in phyla with available structural data on *bd* oxidases, i.e., Actinobacteria, Firmicutes, and Proteobacteria, the structural core consists of two large subunits with an invariant fold, denoted as CydA (approximately 60 kDa) and CydB (approximately 40 kDa) in most *bd* oxidases and as AppC and AppB in the second *bd* oxidase (*bd*-II) in *E. coli*. These subunits are arranged in a two-fold rotational pseudo-symmetry, with both subunits comprising nine transmembrane (TM) helices each. These nine TM helices are arranged in two membrane-spanning four-helix bundles and an additional peripheral transmembrane (TM) helix (Figure 1). Beyond this, all three heme groups, denoted as hemes *b*_558_, *b*_595_, and heme *d*, are located in CydA (or AppC, respectively). They are arranged in a triangle with two heme groups oriented linearly in a plane and the third one sitting orthogonally above this plane (Figure 2A,B). Despite its structural homology to CydA/AppC, the second large subunit CydB/AppB does not carry any metal-containing cofactors. Instead, for CydB of *E. coli bd*-I and AppB of *E. coli bd*-II, it has been found that a ubiquinone-8 or demethylmenaqinone-8 occupies a position equivalent to the heme binding sites in CydA/AppC (Figure 2C) [10,11,14,15], perfectly tracing the positions of the hemes in the opposing subunit. Hence, it has been postulated that this quinone has mainly stabilizing properties. This assumption is supported by the fact that mutations of amino acid residues in the ubiquinol-binding groove of *E. coli* CydB lead to a loss of activity [10]. The quinol must be very tightly bound to CydB because ubiquinone is not displaced by a high excess of aurachin D, a specific quinol-site inhibitor [10], and demethylmenaquinone is not replaced from AppB by a high excess of menaquinone-8 [15]. However, the presence of demethylmenaquinone-8 in one of the published *E. coli bd*-II structures (pdb ID 7OY2) was additionally suggested to indicate a close association of *bd*-II biosynthesis with menaquinone synthesis pathways [15]. In that study, menaquinones were discussed as co-substrates of *bd*-II in aerobic respiration, when molecular oxygen is limited, thus extending the role of menaquinones in *bd*-II biosynthesis and enzyme catalysis. The observed divergence of the type of quinol found in AppB of *E. coli bd*-II, with binding of ubiquinol in one structure [14] and demethylmenaquinone in the other [15], is most likely due to different conditions of cell growth and protein production. Importantly, the *bd* oxidases of Mycobacteria lack these quinones bound to CydB [12,13]. Instead, they contain multiple tryptophan and phenylalanine residues that take this place, creating a stabilizing network of van-der-Waals contacts and making quinone binding redundant (Figure 2D).

**Figure 3:**
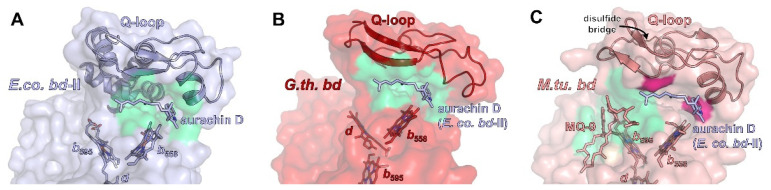
Quinol binding sites in bacterial *bd* oxidases. (**A**) Quinol binding site in *E. coli bd*-II (pdb ID 7OSE). The specific inhibitor aurachin D is shown as sticks, the interacting surface of CydA is shown in green, the residual protein surface in light blue. The Q-loop is involved in binding and provides the top half of the binding site. (**B**) Aurachin D (modelled from 7OSE after superimposing 5DOQ and 7OSE) perfectly fits to the putative quinol binding site in *G. thermodenitrificans* (5DOQ, green surface, residual protein surface given in red). (**C**) The corresponding cleft below the Q-loop in mycobacterial *bd* oxidases (shown for *M. tuberculosis*) is too narrow for aurachin D (putative clashes shown in hotpink). Instead, a quinol binding site was identified close to heme *b*_595_, where menaquinone-9 was found to interact with CydA (green surface, residual protein surface in light salmon).

All *bd* oxidases feature an additional structural element that is, in most cases, crucial for substrate binding, that being the Q loop [16,86]. It is inserted between TM helix 6 and 7 of CydA/AppC in close proximity to heme *b*_558_ (Figure 3). Accordingly, it has been proposed that the Q-loop is involved in the binding of the quinol substrate (from which the name is derived). The N-terminal region of the Q-loop shows a higher amount of conserved amino acid residues than the C-terminal portion [87,88]. Hydrogen/deuterium exchange mass spectrometry measurements demonstrated that the common N-terminal domain of the Q-loop is intrinsically flexible [11], while its C-terminal extension present in the members of the L-subfamily is rather rigid and extends all over the periplasmic surface of CydA to the CydAB interface [10,11]. This implies a functional role for the N-terminal domain of the Q-loop and a structural role for its C-terminal extension. These differences might explain why exchanging the Q-loop between various forms of *bd* oxidases is not possible, with the only exception being the Q-loop of *E. coli bd*-II that may replace that of *E. coli bd*-I [89,90]. Importantly, *bd* oxidase from *M. tuberculosis* and *M. smegmatis* are usually classified as belonging to the S-subfamily [12]. However, they contain an extra insertion of eight amino acid residues in a periplasmic loop of CydA, which represents a rigid unit [13]. These extra residues interact with a small helix called Q*h3* of the C-terminal domain of the Q-loop via a cluster of hydrophobic amino acid residues [13]. It is known that some of these residues are essential for the activity of the mycobacterial enzyme [91]. This arrangement is further stabilized by several H-bonds, implying that these interactions are most likely not involved in quinol oxidation. Furthermore, the N-terminal part of the mycobacterial Q-loop is fixed by a unique disulfide bond. Altogether, the distinctive flexibility of the Q-loop that is expected to be essential for its function in quinol oxidation in Proteobacteria is drastically diminished in the mycobacterial enzyme. Thus, the restricted conformational dynamics of the Q-loop most likely prevents the binding of quinol (Figure 3) [13].

Although at least parts of the Q-loop were successfully modelled in all available structures, only for a single member of the L-subfamily, *E. coli bd*-II, has the Q-loop been structurally resolved, and this is only because aurachin D was added, which fixated the flexible loop in a defined conformation [14]. However, the structural work available to date shows that the lid-like position on subunit CydA/AppC on the periplasmic or extracellular side seems to be a general feature of the Q-loop over all phyla (Figure 3).

### 2.2. Additional Subunits

**Figure 4:**
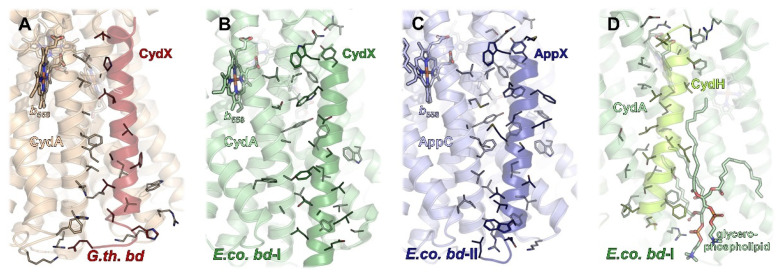
Interaction interfaces of additional subunits with the *bd* core subunit CydA/AppC. (**A**–**C**) Subunit CydX/AppX (bold colours) binds to subunit CydA/AppC (light colours) in a largely conserved position close to heme *b*_558_, lateral to the Q binding site (not shown). Interactions are mainly driven by hydrophobic contacts. (**A**) Interaction patterns between CydX and CydA in *G. thermodenitrificans bd*. (**B**,**C**) CydX/AppX of *E. coli bd*-I and *bd*-II bind in a nearly identical manner to CydA/AppC, underlining the close homology of both *bd* oxidases. As compared to *G. thermodenitrificans bd*, the N-terminus (top) of CydX/AppX is tilted further away from heme *b*_558_. (**D**) Subunit CydH (limon), exclusive to *E. coli bd*-I, is found at the opposite side of CydA (light green) and appears to be further stabilised by a glycerophospholipid (shown as sticks). Again, mainly hydrophobic interactions contribute to binding.

Proteobacterial cytochrome *bd* oxidases contain at least one additional single-helix TM subunit [92,93] that was shown to be crucial for the assembly, stability, and activity of the enzyme [93]. In *G. thermodenitrificans bd* oxidase, this subunit (approximately 4 kDa) is called CydS, while in *E. coli*, it has been denoted as CydX (*bd*-I) or AppX (*bd*-II), respectively. It was found to be oriented alongside the TM helices of CydA/AppC and to bind to that subunit through numerous interactions with TMs 1, 4, 5, 6, and 7 (Figure 4A–C). The intricate interaction pattern appears to be the cause for the requirement of CydS/CydX/AppX for the stable assembly and activity of proteobacterial *bd* oxidases. In one of the cryo-EM structures, *bd*-II reconstituted into amphipols is mainly present as a dimeric species [14]. Dimerization is mediated by several weak hydrophobic interactions between the small AppX subunits. It is noteworthy that none of the other structures reported a dimeric version of a *bd* oxidase. However, the ability of *E. coli bd*-II to dimerize does not change the overall position of AppX with respect to AppC as compared to the homologous pair of CydX/CydA in *E. coli bd*-I, nor does it affect any of the protein-protein interactions. Interestingly, the homologous subunit CydS in *G. thermodenitrificans* is slightly tilted towards TM6 of CydA (Figure 4A), resulting in a maximum shift of 4–5 Å at the periplasmic side. Although this tilt excludes TM4 from binding, the general function of CydS was suggested to be equivalent to the *E. coli bd* oxidases [9].

In addition to CydX/CydS/AppX, *E. coli bd*-I oxidase contains another single-helix subunit called either CydY or CydH [10,11]. This small subunit (approximately 3 kDa) binds in a cleft between TM helices 1 and 9 of CydA (Figure 4D). This subunit is encoded by the former orphan gene *ynhF* that is neither part of the *cyd* nor the *app* operon encoding both *E. coli bd* oxidases. Homologues of this subunit have previously been identified in the proteobacterial clade, where they coincide with the presence of the L-subfamily *bd* oxidases [11]. In contrast to CydX/CydS/AppX that mainly seems to play a structural role, CydY/CydH appears to be of functional importance, as it blocks the putative oxygen access site of *G. thermodenitrificans bd* oxidase (see below). This already implies a different oxygen entry to the *E. coli* and the *G. thermodenitrificans bd* oxidases.

### 2.3. Quinol Binding Site(s)

The substrate quinol is usually bound and oxidized at the interface of the Q-loop and TM helices 6 and 7 of CydA. It has been established that the Q-loop fulfils a critical function in the structural arrangement of the propionate group of heme *b*_558_ relative to the quinol [87]. In addition, the Q-loop contains a conserved glutamate and lysine residue that are essentially involved in the binding and oxidation of the quinol [87]. The high flexibility of the N-terminal part of the Q-loop is most likely needed for the fast and transient binding of the quinol. So far, there is no structure of a *bd* oxidase with a quinol tightly bound to the Q-loop, which is most likely due to its extremely dynamic binding. Several attempts were made to obtain a structure with a bound Q-site inhibitor or a bound quinone by incubating the enzyme preparation with a huge molar excess of aurachins, quinolone-type inhibitors, and ubiquinone and menaquinone [10,11,15]. However, none of the substances gave rise to an additional electron density close to the Q-loop after cryo-EM. The only exception is the binding of aurachin D to *E. coli bd*-II oxidase [14]. Here, an extra electron density was detected that unambiguously derived from aurachin D (Figure 3A). This might be due to the extremely high affinity of aurachin D to this oxidase of only about 7 nM [14] and, thus, the only available inhibitor with an IC_50_ below 10 nM. The addressed protein surface features a pronounced complementarity to the inhibitor. Binding is mostly mediated by hydrophobic interactions, but a single H-bond to an aspartate, which is conserved within Proteobacteria but not in Mycobacteria, adds to the strong binding. Mutation of this single residue to an asparagine residue led to a more than fivefold decrease of the IC_50_ and diminished the enzyme activity to one fourth [14]. Unexpectedly, a menaquinone-9 molecule in its oxidized form was detected in the unusual Q-loop of the *M. tuberculosis* oxidase close to the unique disulfide bond (Figure 3C) [13]. This interaction is further supported by the porphyrin scaffold of heme *b*_595_ and several residues from TM helices 1 and 9 [13]. The naphtoquinone headgroup interacts with a methionine residue close to the extra eight amino acid residues inserted in a periplasmic loop of CydA and a tryptophan residue at the N-terminus of CydA. These residues are only conserved among Myobacteria. It is noteworthy that this menaquinone binding site is fully occupied by CydY/CydH in *E. coli bd* oxidases. Molecular dynamic (MD) simulations imply that either an oxidized or a reduced menaquinone may bind at this position [13]. Thus, it seems that in Mycobacteria, the Q-loop is structurally fixed and incapable of quinol binding and reduction, while the open access to heme *b*_595_ might be used as an alternative electron pathway leading directly to heme *d*, thus bypassing heme *b*_558_ [13].

### 2.4. Heme Arrangement and Electron Transfer

Despite their common architecture and although the heme binding sites themselves are conserved in all available structures, *bd* oxidases further differ in the heme arrangement within the triangle (Figure 2A,B). While the low-spin heme *b*_558_, the first of the two hemes arranged on a plane, is always found closest to the Q-binding site near the Q-loop and its axial ligands, histidine and methionine are conserved, and the second and the third heme groups, high-spin hemes *b*_595_ and *d*, are located at different positions within the triangle depending on the species.

The *bd* oxidase from *G. thermodenitrificans* harbors heme *b*_595_ in the plane of heme *b*_558_ [9]. Here, both axial coordination sites of the central Fe-atom are occupied by histidine and glutamate residues (Figure 2A). The glutamate residue homes in onto the Fe atom from the distal side of heme *b*_558_ and the Q-binding site to fully occupy the axial ligand site at an O-Fe distance of 2.1 Å. Oxygen, the terminal electron acceptor, is prohibited any access to that heme. The orthogonally placed heme *d*, however, does feature an oxygen cavity lined by a threonine and a leucine residue. Heme *d* is situated closely enough to the protein-membrane interface to allow for rapid diffusion of oxygen from the membrane. Hence, this heme most likely is the site where molecular oxygen is reduced to water.

Unexpectedly, the heme orthogonally placed to heme *b*_558_, which is heme *d* in *G. thermodenitrificans* [9], is heme *b*_595_ in all other structures [10,11,12,13,14,15]. Again, the strictly conserved glutamate residue serves as an axial ligand proximal to heme *b*_558_ (Figure 2B) and, equivalent to *G. thermodenitrificans* heme *d*, the distal coordination site of the Fe atom is always found unoccupied. However, in all species harbouring heme *b*_595_ at this position, a phenylalanine approaches the coordination site to a distance of 3.4 Å, enough for hydrophobic contacts to the porphyrin moiety to occur, but too little for binding of molecular oxygen. Hence, heme *b*_595_ found in that position is not the dioxygen reduction site. Instead, this is heme *d*, coordinated by the well conserved histidine residue as first axial ligand proximal to heme *b*_558_ (Figure 2B) [10,11,12,13,14,15]. The distal side of heme *d*, however, does not have a proteinaceous ligand, but was found to be either unoccupied or to contain an extra electron density that was interpreted as molecular oxygen [10,11,12,13,14,15]. Here, the coordinating glutamate residue is located more remotely at a distance of at least 4.8 Å to the central Fe atom. The shift of the coordinating glutamate originates from an inserted leucine residue (Leu101 in *E. coli bd*-I and *bd*-II and *M. smegmatis bd;* Leu100 in *bd* of *M. tuberculosis*; this residue is lacking in *G. thermodenitrificans bd* oxidase) into TM helix 3 of CydA, resulting in its stronger curvature. This generates a voluminous cavity for the binding of dioxygen at the axial position of heme *d*, which is enclosed by hydrophobic isoleucine and phenylalanine residues (Figure 2B). Thus, an ideal environment for binding a dioxygen molecule is provided. Since this site is buried deeply inside the enzyme core, it requires an oxygen channel, and indeed, such a channel leads straight from the membrane through to heme *d* (see below).

For the reduction of dioxygen to water, four protons and four electrons are needed. From the triangular arrangement of and the distances between the hemes in *bd* oxidases, conclusions were drawn on the electron transfer and the mechanism of dioxygen reduction. As stated above, the heme arrangement in *G. thermodenitrificans* differs from that in all other *bd* oxidases. Here, the short distance between heme *b*_558_ close to the quinol binding site and the active site heme *d* implies a direct electron transfer from *b*_558_ to *d*. The electron would then equilibrate between the *d* and the *b*_595_ hemes [9]. In the reduced enzyme, two electrons may derive from heme *d* via the oxoferryl-state, one from *b*_595_ and the fourth electron from the macrocycle of heme *d* [9,23,24]. This very rapid or simultaneous four-electron transfer mechanism is reminiscent of that of HCOs preventing the formation of reactive oxygen species [9,23]. The other *bd* oxidases feature a short distance between the two *b*-type hemes, implying a sequential electron transfer from *b*_558_ via *b*_595_ to heme *d*. Reduction of dioxygen is then catalyzed by a short-lived peroxide intermediate in agreement with spectroscopic data [10,11,19,23].

### 2.5. Oxygen Access

**Figure 5:**
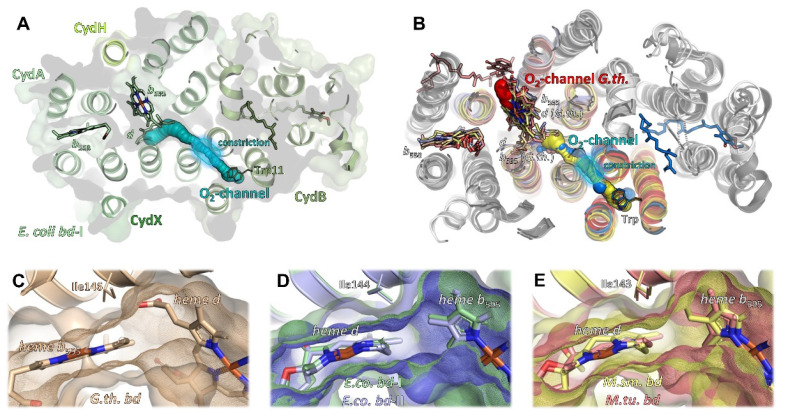
Oxygen channels to the active site in bacterial *bd* oxidases. (**A**) Oxygen channel in *E. coli bd*-I (cyan) leading from Trp11 in CydB through the protein core to heme *d*. The channel features a constriction that is thought to serve as selectivity filter for linear molecules such as dioxygen. (**B**) Other *bd* oxidases feature an equivalent oxygen channel (*E. coli bd*-II in marine blue, *M. smegmatis bd* in yellow, *M. tuberculosis bd* in salmon) with a similar constriction. Only *G. thermodenitrificans bd*, as a consequence of the altered arrangement of the heme groups, features a very short channel (red) from the opposing side of the enzyme and leading directly to the active site heme *d*. (**C**–**E**) A conserved isoleucine residue (shown as thick lines) blocks diffusion of dioxygen between hemes *b*_595_ and *d* by perfect surface complementarity. Hemes are given as sticks, protein surfaces (smooth surfaces) and surfaces of hemes (meshes) are provided to illustrate the excellent surface match. (**C**) *G. thermodenitrificans bd*, (**D**) *E. coli bd*-I (pale green) and *E. coli bd*-II (slate blue), (**E**) *M. smegmatis* (pale yellow) and *M. tuberculosis bd* (salmon).

The *bd* oxidases from *M. tuberculosis* and *M. smegmatis* consist only of subunits CydA and CydB [12,13]. Although it cannot be completely excluded that additional small subunits being part of these oxidases are encoded in the respective genomes, no orphan genes have been identified in the mycobacterial databases that may code for such subunits [13]. Furthermore, these oxidases are fully assembled, stable and catalytically active, although they do not contain another subunit [12,13]. But while in *E. coli bd*-I oxidase, oxygen access to heme *b*_595_ is blocked by CydY/CydH (Figure 4C) [10,11], and, instead, molecular oxygen is directed through a hydrophobic channel directly to heme *d* (Figure 5A), *E. coli bd*-II oxidase and the mycobacterial *bd* oxidases, which do not have such a subunit, rely on an intrinsic barrier that is present in all *bd* oxidases, an isoleucine residue (Ile143 in *M. smegmatis* and *M. tuberculosis*, Ile144 in *E. coli bd*-I and *bd*-II, Ile146 in *G. thermodenitrificans*, Figure 5C–E). Hence, although heme *b*_595_ is freely accessible from the membrane due to the lack of a small subunit, all enzymes with the exception of that from *G. thermodenitrificans* utilize an oxygen channel, leading from CydB to heme *d* bound to CydA (Figure 5B), just as described for *E. coli bd*-I [10,11,14,15]. Accordingly, this small hydrophobic channel, always starting above a conserved tryptophan residue on CydB/AppB and extending further to CydA/AppC, is a common feature of all oxidases except for the one from *G. thermodenitrificans*. Common to these oxygen channels is a constriction that may act as a selectivity filter disabling the passage of angled molecules. The small channel leading directly to heme *b*_595_ was proposed as a second oxygen access channel in the structure of the *M. smegmatis bd* oxidase [12]. However, experimental evidence for this hypothesis has not yet been provided. It is worthy of note that this is the position for which it is assumed that it binds menaquinol in *M. tuberculosis bd* oxidase (see above) [13]. As opposed to this, oxygen access to *bd* oxidase from *G. thermodenitrificans* is principally different due to the re-arrangement of the *b*_595_ and *d* heme groups [9,10,11]. Here, heme *d* is freely accessible from the membrane via a very short channel, making an additional channel as found in all other oxidases superfluous (Figure 5B).

### 2.6. Proton Pathways

**Figure 6:**
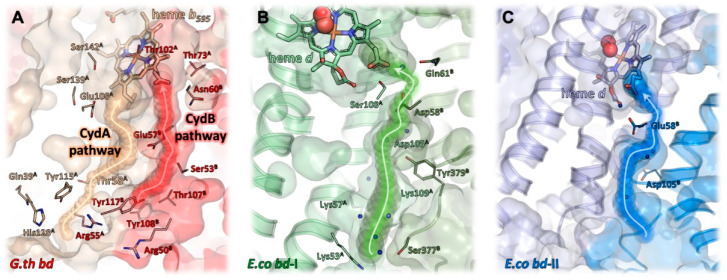
Proton pathways in bacterial *bd* oxidases to the heme triangle. (**A**) *G. thermodenitrificans* features a pathway along the CydA/CydB interface, termed the CydB pathway (red). A second pathway running through CydA has been proposed as well, termed CydA pathway (light beige). (**B**) A pathway in *E. coli bd*-I (olive green), largely equivalent to the CydB pathway in *G. thermodenitrificans*, is lined by several hydrophilic amino acid sidechains (shown as thick lines) and directs water molecules (i.e., protons) to the propionate of the active site heme *d*. Numerous water molecules (blue spheres) have been found in that channel, highlighting its full accessibility. (**C**) *E. coli bd*-II features a comparable channel. But due to the much wider cavity protruding deeply into the protein core, the actual channel requires a only a glutamate and an aspartate sidechain (shown as sticks) to coordinate water molecules and guide them to the active site. Here again, the presence of water molecules illustrates the accessibility of solvent molecules.

In addition to electrons delivered from quinol, the reduction of dioxygen to water requires protons. As the reaction of *bd* oxidases is electrogenic [8], protons for dioxygen reduction are taken up at the cytosolic side of the membrane, while the protons generated during quinol oxidation are released to the periplasmic side of the membrane. To enable a fast oxygen reduction, the oxygen channel and the proton pathway(s) should meet at the open coordination side of heme *d*. It was proposed that the oxygen channel may also conduct protons by a connection with a proton pathway [94]. In *G. thermodenitrifcans bd* oxidase, the proton pathway leads to heme *b*_595_, and from there the protons are most likely further transferred to the *d* heme [9]. That implies that all proton pathways lead to the heme group that is located the closest to the cytosol. And indeed, the proton pathways in the various *bd* oxidases are very similar to each other and start at a broad and shallow hydrophilic cavity at the cytosolic side of the CydAB/AppCB interface [10,11,12,13,14,15]. This cavity narrows to a channel of about 4 Å diameter and runs perpendicular to the membrane, flanked by the TM helices 2 and 3 of CydA/AppC and CydB/AppB, respectively (Figure 6). A series of serine, glutamate and aspartate residues and several water molecules lead directly to the propionate group of heme *d* [10,11,12,13,14,15]. Remarkably, this proton pathway splits up to create a second branch that leads to the oxygen channel in CydB/AppB. This branch ends at a conserved aspartate residue that is essential for the activity of *E. coli bd*-I, a member of the L-subfamily of *bd* oxidases [95]. Due to the large distance of 20 Å of this residue to heme *d*, its direct participation in dioxygen reduction is unlikely. It rather acts as proton storage or is required for charge compensation [15]. An additional solvent-accessible area was detected on the periplasmic side of the *M. tuberculosis* oxidase by MD simulations [13]. Here, water molecules located between the TM helices 5 and 6 of CydA connect the propionate group of *b*_558_ with a conserved glutamate residue close to *b*_595_ over a distance of about 12 Å. It was proposed that this solvent filled area functions as a “dielectric well” and may facilitate charge compensation [13] as expected for the second branch of the proton pathway from the cytoplasmic side described above. For the *G. thermodenitrificans* oxidase, two different proton pathways were proposed [9]. One is located in the four-helix bundle formed by the TM helices 1–4 of CydA and the second one in the symmetry-related helices in CydB [9]. Accordingly, they have been named CydA and CydB pathways. Both pathways lead to heme *b*_595_ with the CydA pathway ending directly at the conserved glutamate, the ligand of *b*_595_. However, the route of the additional proton transfer to heme *d* needed for the reaction of dioxygen to water is still unclear [9]. Future studies will have to address this point by means of the site-directed mutagenesis of putatively involved sidechains. In addition, recent advances in the field of cryo-EM may make high-resolution structures available that will support a refined identification and characterization of water, proton, and oxygen pathways and in particular the involved protein sidechains. It is anticipated that the upcoming years will bring about a wealth of novel data, which will deepen our understanding of the catalytic principles of *bd* oxidases.

## 3. Cytochrome *bd* as a Prospective Target for the Development of New Antibiotics

When bacterial pathogens colonize host cells and tissues, they encounter adverse environmental conditions, such as hypoxia or the presence of reactive oxygen and nitrogen species generated by the host immune system as weapons against the invaders. An increase in the cytochrome *bd* expression is a mechanism for survival used by the pathogenic microorganisms under these conditions. This is likely due to the unique structural features of the *bd* oxidase, which allows it to function actively in these and other unfavorable environments. Accordingly, the promotion of virulence by cytochrome *bd* was observed in *Listeria monocytogenes*, *M. tuberculosis*, uropathogenic *E. coli* (UPEC), *Shigella* sp., Group B *Streptococcus*, *Salmonella enterica* serovar Typhimurium, *Clostridia* species, *Staphylococcus aureus*, and *Burkholderia pseudomallei* [52,96,97]. The importance of cytochrome *bd* for pathogenic bacteria justifies its choice as a potential drug target.

Particularly noteworthy is the fact that the *bd* oxidase protects mycobacteria from antibiotic stress by allowing them to respire. *M. tuberculosis* is the causative agent of tuberculosis, the 13th leading cause of death, and the second leading infectious killer after COVID-19 [98]. Most troubling is the continued rise in drug-resistant forms of the disease. Multidrug-resistant (MDR), extensively drug-resistant (XDR), and totally drug-resistant (TDR) strains of *M. tuberculosis* pose a serious threat to public health. Thus, new antimicrobials with novel mechanisms of action are urgently needed.

The *M. tuberculosis* electron transport chain has recently gained interest as a target space for next-generation antibacterials [91,99,100,101,102,103,104,105,106,107,108,109]. *M. tuberculosis* possesses a branched aerobic respiratory chain. The menaquinone pool receives electrons from various dehydrogenases including one proton-pumping NADH dehydrogenase, Nuo (type I NDH-1), two non-proton-pumping NADH dehydrogenases, Ndh and NdhA (type II NDH-2), and two succinate dehydrogenases, SDH-1 and SDH-2 [110]. The electrons from the reduced menaquinone (menaquinol) can then be transferred to O_2_ via two different terminal respiratory enzymes. Usually, the choice depends on the oxygen tension. Under aerobic conditions, this is a *bcc*-*aa*_3_ supercomplex composed of cytochrome *bcc* (complex III or qcrBCD) and the *aa*_3_-type cytochrome *c* oxidase (complex IV or ctaBCDE). Under low oxygen concentrations, cytochrome *bd* (cydAB) serves as a terminal oxidase [111]. Each of the terminal segments of the respiratory chain generates a proton motive force, albeit with different efficiency, to drive ATP synthesis via the F_1_F_o_-ATP synthase (atpBEFHAGDC).

Pharmacological targeting of the mycobacterial respiratory enzymes holds significant clinical promise. The first antimycobacterial drug of this kind approved by the FDA and EMA is bedaquiline (Sirturo™). The drug selectively inhibits the F_1_F_o_-ATP synthase [112]. The other compound that targets *M. tuberculosis* cellular energy production is Q203 (telacebec). The compound targets the cytochrome *b* subunit (QcrB) of cytochrome *bcc* [113]. Q203 has gone through three clinical studies, the most recent being a phase 2a efficacy trial [114]. Cytochrome *bd* appears to contribute to the defense of mycobacteria against the stress induced by each of the two antibiotics. The defense mechanisms seem to be different. In the case of bedaquiline, the *bd* oxidase possibly detoxifies reactive oxygen species generated by the drug. As to Q203, cytochrome *bd* provides an efficient alternate respiratory route for electrons transferring from menaquinol to O_2_ (see [53] and references therein). Accordingly, genetic or chemical inhibition of the *bd* oxidase has synthetic lethal interactions in *M. tuberculosis* with the bacteriostatic Q203, leading to rapid cell death against both replicating and non-replicating cells in vitro and in a mouse model of tuberculosis [115,116]. These findings raise hopes that combinations of respiratory chain inhibitors, which include a cytochrome *bd* inhibitor, may have a rapid and high killing capacity towards *M. tuberculosis* and other pathogens containing the *bd* oxidase.

**Figure 7:**
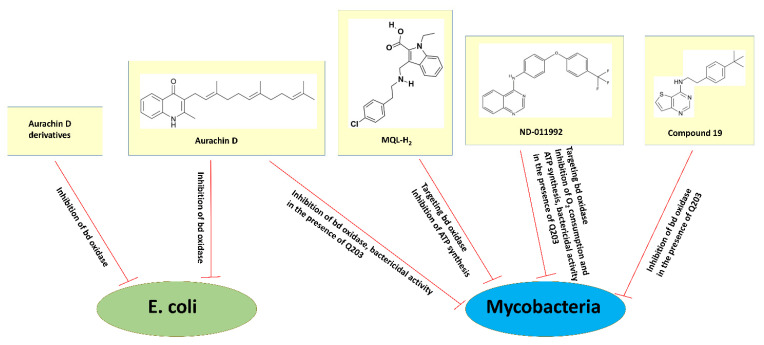
Simplified schematic representation of the effects of compounds targeting the *bd* oxidase on the level of the isolated enzyme, membrane vesicles or whole cells. Shown are the structures of aurachin D, 3-[[2-(4-chlorophenyl)ethylamino]methyl]-1-ethyl-indole-2-carboxylic acid (MQL-H_2_), *N*-(4-(4-(trifluoromethyl)phenoxy)phenyl)quinazolin-4-amine (ND-011992), and *N*-(4-(*tert*-butyl)phenethyl)thieno [3,2-*d*]pyrimidin-4-amine (Compound **19**). See the main text for details. Data collected from: [10,13,14,116,117,118,119,120,121,122,123,124].

The search for cytochrome *bd* inhibitors suitable for clinical purposes is at the very beginning. The quinone-analogs aurachins and their derivatives have attracted particular attention. Aurachins are isoprenoid quinoline alkaloids originally extracted from myxobacteria. Meunier et al. first reported that aurachin C and aurachin D are powerful inhibitors of the terminal quinol oxidases of *E. coli* [117]. The addition of 214 nM aurachin C to the *E. coli* membranes containing either cytochrome *bd*-I (strains GL101 or GL105) or the heme-copper cytochrome *bo*_3_ (strain RG145) inhibits the duroquinol:O_2_ oxidoreductase activity of each oxidase by 90%. 400 nM aurachin D, in turn, inhibits the membrane-bound *bd*-I by 93% and the membrane-bound *bo*_3_ by as little as 5%. Thus, aurachin C is effective on both quinol oxidases, whereas aurachin D displays selectivity for inhibition of cytochrome *bd*-I [117] (see also Figure 7). Theßeling et al. determined the apparent IC_50_ of aurachin C and aurachin D towards the duroquinol:O_2_ oxidoreductase activity of the isolated cytochrome *bd*-I from *E. coli* (strain BL21 Δ*cyo*/pET28a *cydA_h_BX*) to 12 and 35 nM, respectively [10]. Aurachin C and aurachin D also strongly inhibit the duroquinol:O_2_ oxidoreductase activity of the isolated *E. coli* cytochrome *bd*-II (strain BL21 Δ*cyo*/pET28b(+) *appC_his_BX*) with the apparent IC_50_ of 7.1 and 11.1 nM, respectively [14].

Radloff et al. studied the inhibitory effects of aurachin C and new aurachin D derivatives on the ubiquinol-1:O_2_ oxidoreductase activity of the isolated cytochromes *bd*-I, *bd*-II, and *bo*_3_ from *E. coli* (strains C43 Δ*bo*_3_/pET17b-*cydABX*-StrepII, C43 Δ*bo*_3_/pET17b-*appCBX*-StrepII, and GO195 pIRHisA, respectively) [118]. Long- (C10, decyl or longer) and short-chain (C4, butyl to C8, octyl) aurachin D derivatives were synthesized. Their inhibitory potency and selectivity were assessed. The authors confirmed the strong inhibition of all three quinol oxidases by aurachin C derivatives in a nanomolar range and the fact that none of these compounds selectively inhibits a certain oxidase. Earlier data showed that the replacement of N–OH with an N–H group in aurachin C and its analogs decreases the inhibitory potential only for the *bo*_3_ enzyme, keeping a strong inhibitory effect on the *bd*-I enzyme [119]. Correspondingly, all aurachin D derivatives tested clearly show the inhibition of both cytochrome *bd*-I and cytochrome *bd*-II (Figure 7). As the inhibitory effect on the *bd*-I enzyme was higher than that on the *bd*-II enzyme, the former was investigated in most experiments. Two short-chain aurachin D derivatives, 2-(2-heptyl)-3-methyl-4(1H)-quinolone (AD7-1) and unsaturated AD7-1 with a double-bond in the heptyl-side chain (AD7-1*), were found to be highly selective towards the *bd*-I oxidase. Their apparent K_i_ values appeared to be similar to that of the natural aurachin D. Furthermore, the inhibitory activity was shown to increase with increasing chain length at position R1 of the 2-methyl-4-quinolones backbone. Among the inhibitors tested, AD7-1 combines properties of high inhibitory potency and selectivity for cytochrome *bd*-I while causing no inhibition of cytochrome *bo*_3_ in the low nanomolar to micromolar range. It was concluded that AD7-1 could be the promising candidate for trials on a physiological level [118].

Makarchuk et al. screened a target-focused library of small molecules that contains a set of quinones, naphthoquinones, phenols, quinolones, coumarins, and flavonoids to identify potential inhibitors of cytochrome *bd*-I from *E. coli* (strain BL21 Δ*cyo*/pET28a *cydA_h_BX*) using protein film voltammetry [120]. As such, quinolones with alkyl or iodine substituents in positions C-2 and C-3 were identified. The most active inhibitors were produced by chemical modification of the quinolone core and the introduction of an isoprenyl chain in position C-3. The authors showed that the inhibitory efficiency of these compounds increases from one to two isoprene repetitive units and decreases for longer chains [120].

The effect of aurachin D on the activity of the *bd* oxidase in mycobacteria was also investigated. It was shown that the compound inhibits O_2_ consumption of cytochrome *bd* in inverted membrane vesicles of *M. smegmatis* (strain mc^2^155 Δ*qcrCAB*::hyg) with an IC_50_ of ~400 nM [121] (Figure 7). In these experiments, the reaction was started by the addition of NADH as the electron donor. Accordingly, under similar conditions, aurachin D decreases the O_2_ consumption rates of the *bd* oxidase (IC_50_ of 0.158 μM) in inverted membrane vesicles of *M. tuberculosis* recombinantly produced in a *M. smegmatis* mc^2^155 Δ*cydAB* strain [13]. Although a significant inhibitory effect of aurachin D was shown for the membrane vesicles, the compound when applied alone does not effectively inhibit mycobacterial growth. The minimal inhibitory concentrations (MICs) for inhibition of growth of *M. smegmatis* and *M. tuberculosis* were reported to be >85 μM and >100 μM, respectively [121,122]. This finding indicates that aurachin D is not able to efficiently permeate the mycobacterial cell wall [121]. However, the presence of aurachin D significantly enhances the growth inhibition of *M. tuberculosis* (strain H37Rv) by Q203, a selective inhibitor of cytochrome *bcc* [122] (Figure 7). The Q203 MIC decreases from 10 nM when used alone to 1.25 nM when in combination with 25 μg/mL aurachin D. Furthermore, the addition of aurachin D to *M. tuberculosis* H37Rv converts the bacteriostatic activity of Q203 (30 × MIC) into bactericidal activity in a dose-dependent fashion [122]. Consistently, the almost identical bactericidal activity of Q203 was observed within cytochrome *bd* knockout strains [122]. Thus, the *bd* oxidase inhibitor aurachin D can considerably stimulate the impact of a companion drug targeting cytochrome *bcc*.

Harikishore et al. employed an in silico screening to identify a novel inhibitor 3-[[2-(4-chlorophenyl)ethylamino]methyl]-1-ethyl-indole-2-carboxylic acid (MQL-H_2_) that likely targets mycobacterial cytochrome *bd* at the menaquinol binding site [123]. The compound was shown to inhibit the ATP synthesis of inverted membrane vesicles of the wild-type *M. smegmatis* (strain mc^2^155) driven by both NADH and succinate with IC_50_ of 60 and 75 μM, respectively (Figure 7). Similarly, the addition of MQL-H_2_ causes the inhibition of NADH-driven ATP synthesis in inverted membrane vesicles of the cytochrome *bcc* deficient *M. smegmatis* mutant (strain mc^2^155 Δ*bcc*) with an IC_50_ of 34 μM. These new data may pave the way for medicinal chemistry-based hit optimization efforts of MQL-H_2_.

Lee et al. identified a small molecule, *N*-(4-(4-(trifluoromethyl)phenoxy)phenyl)quinazolin-4-amine (ND-011992), that seems to target cytochrome *bd* [116]. ND-011992 is ineffective on its own, however, its combination with Q203 inhibits O_2_ consumption and ATP synthesis in *M. tuberculosis* H37Rv and *M. bovis* BCG (Figure 7). The addition of ND-011992 to Q203 does not accelerate the frequency of spontaneous resistant mutations in the pathogen. The ND-011992/Q203 combination appeared to be bactericidal against clinical isolates of various phylogenetic lineages, against MDR and XDR isolates, and kills replicating and antibiotic-tolerant non-replicating mycobacteria in vitro. Furthermore, Q203 being supplemented with ND-011992 achieves better killing than Q203 alone in a mouse model of tuberculosis infection. Although the in vivo study shows that the presence of ND-011992 enhances the potency of Q203, the enhanced potency is limited by ND-011992′s less-than-optimal pharmacokinetic properties. Lead optimization is clearly needed to optimize the potency of the compound and improve its pharmacokinetic properties [116]. In general, these data suggest that inhibitors of the terminal oxidases could be part of a short sterilizing drug combination for tuberculosis.

Hopfner et al. found that another class of compounds, thieno[3,2-*d*]pyrimidin-4-amines, can inhibit cytochrome *bd* in mycobacteria [124]. The authors reported an initial structure-activity-relationship of 13 compounds in *M. bovis* BCG, *M. tuberculosis* H37Rv, and *M. tuberculosis* clinical isolate N0145 in the measurements of ATP depletion in the presence and absence of Q203. All compounds reveal activity against *M. bovis* BCG and *M. tuberculosis* N0145 with ATP IC_50_ from 6 to 54 μM, as determined by ATP depletion in the presence of Q203 (Figure 7). None of the compounds alone inhibit ATP. This clearly suggests that they do target the *bd* oxidase, since potency is observed only if cytochrome *bcc* is selectively blocked by Q203. The tested compounds turned out to be much less potent against *M. tuberculosis* H37Rv as compared to *M. tuberculosis* N0145: the ATP IC_50_ went from 24 to >100 μM vs. 9–52 μM, respectively. A lower potency of the inhibitors in the case of *M. tuberculosis* H37Rv may be due to the increased level of cytochrome *bd* expression in this laboratory-adapted strain. *N*-(4-(*tert*-butyl)phenethyl)thieno[3,2-*d*]pyrimidin-4-amine (named compound **19**) appeared to be the most potent compound, having ATP IC_50_ of 6 to 18 μM against all three strains in the presence of Q203. Based on their results, the authors will aim to develop new members of this class of compounds with improved potency and acceptable pharmacokinetics to warrant in vivo evaluation [124].

## 4. Concluding Remarks

New structural information on cytochrome *bd* should be very helpful for the design and discovery of new antibacterial agents which do not affect the energy metabolism of human organs and tissues. The studies reviewed above show that at present the main efforts are concentrated on the search for inhibitors specific to the cytochrome *bd* quinol oxidation site. We assume that other structural segments of the enzyme, such as the oxygen-binding site, specific intraprotein oxygen channels, and proton transfer pathways, should also be considered as promising targets when creating effective inhibitors of the *bd* oxidase. In general, the development of next-generation antibiotics targeting the respiratory chain enzymes including cytochrome *bd* will contribute to overcoming a great global public health challenge—antibiotic resistance.
